# Label-Free Fluorescent Aptasensor for Small Targets via Displacement of Groove Bound Curcumin Molecules

**DOI:** 10.3390/s19194181

**Published:** 2019-09-26

**Authors:** Baraa J. Alyamani, Omar A. Alsager, Mohammed Zourob

**Affiliations:** 1 National Center for Irradiation Technology, King Abdulaziz City for Science and Technology, P.O. Box 6086, Riyadh 11442, Saudi Arabia; balyamani@kacst.edu.sa; 2 Department of Chemistry, Alfaisal University, Al Zahrawi Street, Al Maather, Al Takhassusi Rd, Riyadh 11533, Saudi Arabia; mzourob@alfaisal.edu; 3 King Faisal Specialist Hospital and Research Center, Zahrawi Street, Al Maather, Riyadh 12713, Saudi Arabia

**Keywords:** aptasensor, curcumin, label-free, fluorescence, vitamin D3, small molecules

## Abstract

Signal transduction based on fluorescence is one of the most common optical aptasensors for small molecules. Sensors with a number of unique features including high sensitivity, low cost, and simple operation can be constructed easily. However, the label-free fluorescent approach is limited to synthetic dyes that bind strongly to the aptamer sequence and result in a diminished sensor operation with high detection limits. In this study, we report the use of curcumin as a fluorescent probe to signal aptamer/small target binding events. A substantial enhancement in curcumin’s fluorescent emission was observed when bound into the grooves of vitamin D_3_ (VTD3) binding aptamer, as an example. However, the introduction of the target molecule causes the aptamer to undergo a conformational change that favors complexing the target molecule over binding the curcumin dye. The sensor was able to detect VTD3 down to 1 fM concentration in buffer solutions and extracted blood samples, operate at a wide dynamic range, and discriminate against potential biological interfering molecules including VTD2. The operation of the curcumin based fluorescent sensor is at least six orders of magnitude more sensitive than a VTD3 sensor constructed with the synthetic dye SYBR Green I. The generality of the reported label-free approach was applied with a previously isolated 75-mer bisphenol-A (BPA) aptamer, confirming that the reported sensing strategy is not confined on a particular aptamer sequence. Our work not only reports a novel sensor format for the detection of small molecules, but also serves fluorescent sensor’s most pressing need being novel fluorophores for multiplex targets detection.

## 1. Introduction

Aptamers are short single stranded oligonucleotides (RNA or DNA) evolved through a combinatorial in vitro chemical process known as systematic evolution of ligands by exponential enrichment (SELEX) [1,2]. Aptamers can sensitively and selectively bind to a wide range of targets including metal ions [3], small molecules [4,5], and large targets [6]. They have shown considerable success in various fields such as analytical sensing, diagnostic, and drug delivery [7,8,9]. Compared with the conventional recognition tools such as antibodies and enzymes in designing biosensors, aptamers exhibit a robust and low production method, low molecular weight, easy modification, and operation in non-immunogenic environments [10]. They are highly regarded as excellent candidates for developing biosensors for targets of medical, environmental, and industrial significance [11]. Due to their reportable molecular conformational change upon target binding, signal transduction have been achieved using electrochemical [12,13], fluorescence [14,15], and colorimetric [16,17,18,19] based platforms.

Particularly, fluorescence is one of the most common optical techniques for aptasensors [20]. Fluorescent aptasensors can be divided into two categories: Labeled and label-free [21]. Labeling aptamers requires a fluorophore and quencher attached to (A) termini of the aptamer in a molecular beacon structure [22,23] and (B) complementary sequences hybridized to the aptamer, such that the fluorophore and quencher are brought to maximum proximity [24,25]. Target recognition induces conformational change in the aptamer structure leading to enhancement or quenching of the fluoresce (case A) or detachment of one or both complementary sequences (case B), resulting in the recovery of the quenched fluorescent signals [26]. Alternatively, the fluorescence of target-free and fluorophore-labeled aptamer can be quenched by the non-specific adsorption into a surface such as; graphene oxide sheets [27], metal organic frame work [28], or nanoparticles [29]. Target recognition leads to the dissociation of the adsorbed aptamer and enhancement of the fluorescent signals. Another approach is to specifically immobilize the aptamer on the surface of particles that act as a quencher for a fluorophore-labeled complementary sequence [30]. Target binding releases the complementary sequence together with the labeled fluorophore resulting in a higher fluorescent signal.

Limitations of labeled aptasensors include that the chemical labeling of aptamer sequences is usually time-consuming, labor-intensive, complex, and cost-ineffective [21]. There is a growing concern that chemical modification of labels or surface adsorption of the aptamers could influence their intrinsic binding affinity and selectivity [31]. Other challenges include that in order to gain maximum signal change upon target binding, the right length and position of the complimentary single stranded (ss)DNA have to be identified, in addition to identifying the exact location of the fluorophore and quencher on the aptamer [24,25,32]. This becomes even very difficult for long aptamers without prior knowledge of the exact location of the binding bucket.

To overcome the abovementioned limitations, label-free fluorescent aptasensors have been developed by duplexing the aptamer sequence with a complementary ssDNA (with or without mismatch) to intercalate specific dyes [33,34]. The formation of aptamer-target complex releases the complementary sequence and allows the construction of signal-on or signal-off aptasensors, depending on the nature of the dye’s interaction and subsequent fluorescent modulation with the double stranded DNA. Although these methods avoid chemical modification of the fluorescent labels, aptamers may still require extension with additional sequences, such that an optimal amount of the aptamer sequence is engaged with the desired complementary sequence [33], and laborious core region mapping steps to achieve efficient strand displacement upon target binding [34].

Developing label-free aptasensors that only use unmodified aptamer sequences to achieve sensitive, selective, and controllable small target recognition sensors remains as an unmet challenge. Very few attempts were made when ssDNA structure selective binding synthetic dyes were used. These dyes include OliGreen (for potassium ions detection using ATP aptamer) [35], SYBER Green I (for ochratoxin A and ATP detection) [36,37], DAPI and Hoechst I (for L-argininamide detection) [38], and Thiazole orange (for tetracycline detection) [39]. A common feature of these fluorophores is that a significant fluorescence change is occurred when they bind to nucleic acid aptamers without regard to base composition. These dyes can be released upon target binding (signal-off sensor) or additional dye chelation (signal-on sensor) is allowed when the conformations of nucleic acid aptamers are changed. High detection limits µM [38] to mM [37] and narrow dynamic range were realized with these sensors, presumably due to the strong binding between the dyes and aptamers. Nevertheless, the detection and quantification of organic molecules in biological and environmental systems is a challenging analytical practice that require detection tools of subnanomolar to high picomolar detection levels, which are not yet met by the previously reported fluorescent sensors [40]. Detection and quantification of organic molecules are routinely conducted by conventional instrumental analysis such as mass spectrometry coupled with chromatography techniques [41]. These systems are expensive, accompanied with a complicated operation, which limit their usefulness in carrying out on-site and real-time detections [42].

**Scheme 1:**
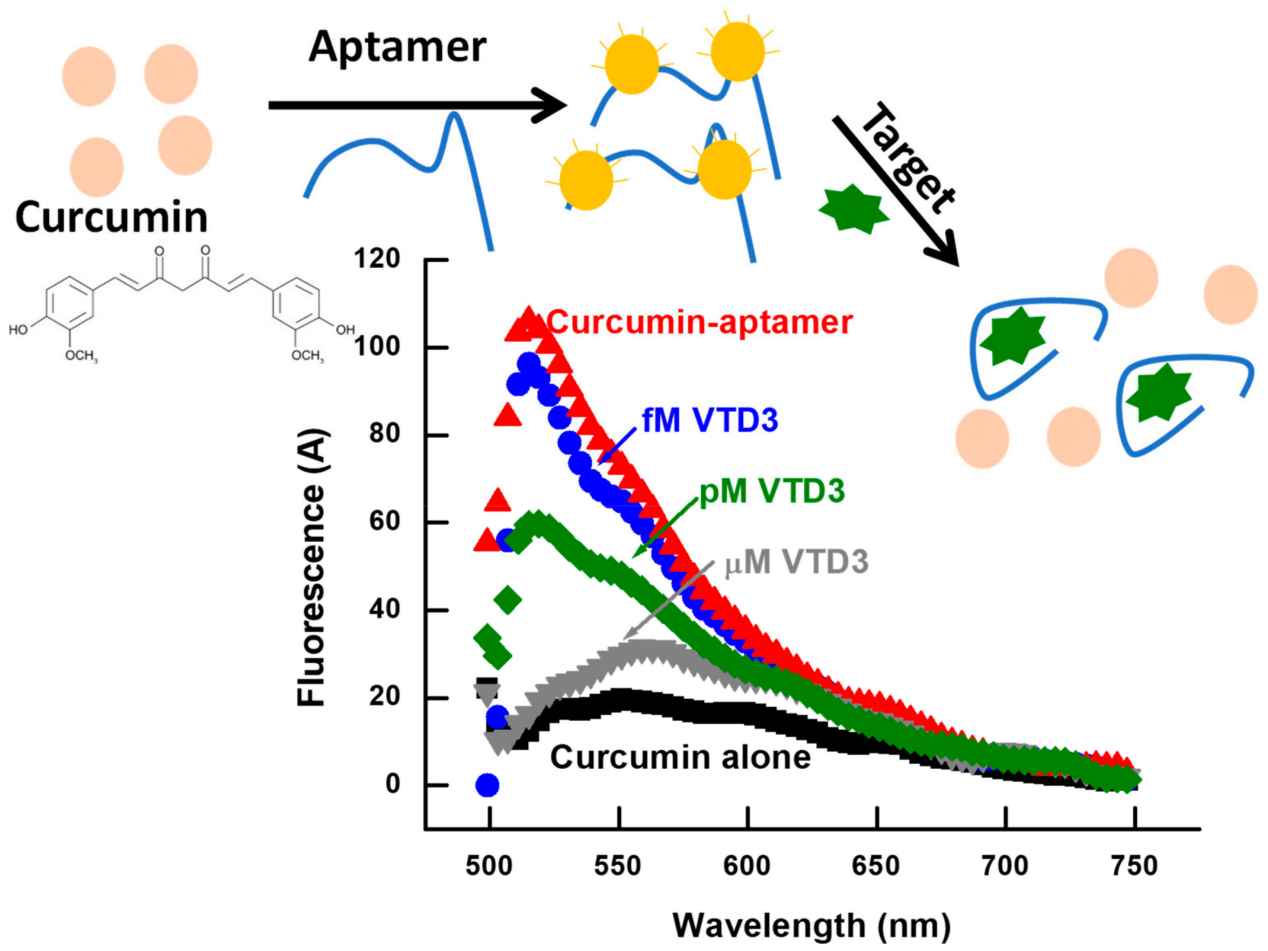
Fluorescence sensor for small molecules with curcumin as a reporter fluorophore. The interaction of curcumin with aptamer enhances the fluorescence intensity. The recognition of the target stimulates the dissociation of the curcumin molecule from the aptamer sequence and induces a subsequent fluorescent quenching, sequentially related to the target concentration (VTD3 in this case).

Herein, we report a novel and general approach to overcome the limitations encountered with the previously applied label-free fluorescent assays, developed mainly with synthetic fluorophores. The naturally occurring dye curcumin was used to transduce aptamers binding to small molecules. The principle of our curcumin based sensor is shown in Scheme 1. Curcumin itself has a limited fluorescence in solution, but shows a strong green emission signal upon binding to the aptamer sequence. Target binding causes conformational change within the aptamer, which leads to the displacement of the weakly bound curcumin molecule. The displacement is accompanied with quenched fluorescent signals that are well correlated with the concentrations of the target molecule. To the best of our knowledge, the reported sensor design achieves the lowest detection limit (1 fM) compared to the previously reported labeled and label-free fluorescent aptasensors for small molecules and all the commercially available VTD3 detection kits. The sensor operates over four orders of magnitude dynamic range, in extracted blood samples, and shows excellent selectivity when VTD3 aptamer was used. The generality of the approach was confirmed with a previously isolated aptamer for BPA. The reported method does not pose any adverse effects on the binding properties of aptamers since no labeling steps of the aptamer or target are required. More broadly, the study provides an effective sensing approach for the growing number of isolated aptamers binding small targets as well as an easily accessible method to characterize ssDNAs post to SELEX selection rounds.

## 2. Experimental Section

### 2.1. Reagents and Chemicals

Vitamin D3 (VTD3) and Vitamin D2 (VTD3) were purchased from Carbosynth Limited, UK. Progesterone (P4), 17β-estradiol (E2), prednisone (PND), and bisphenol-A (BPA) were purchased from Sigma-Aldrich. Curcumin and SYBER Green I were purchased from Alfa Aesar. Deionized water (Milli-Q, 18.2 MΩcm) was used in all experiments (unless otherwise stated), and all other chemicals were of analytical grade. VTD3 aptamer, BPA aptamer, random ssDNA, and VTD3 aptamer complementary sequence were synthesized by Alpha DNA. For the ssDNA preparations, the samples were dissolved in Milli-Q water and kept at −5 °C before use. The sequences of the used aptamers are provided in Table S1 and the secondary structures of the aptamers are presented in the Supporting Information in Figure S1.

### 2.2. Methods

Optimization of reaction conditions for sensing: The optimal conditions for curcumin binding with VTD3 aptamer and BPA aptamer were investigated by titrating curcumin with increasing concentration of the aptamers. Stock solution curcumin was made in ethanol and further dilutions to achieve the desired concentrations were made in deionized water containing 5% ethanol (either having 0.1 M NaCl or non-salted). A 0.6 μM curcumin concentration was titrated with the increasing aptamer concentration (25 nM to 200 nM) in a total reaction volume of 0.5 mL and at two different temperatures, 5 °C and 25 °C. 2 μL aliquots were taken to measure the fluorescence using Nanodrop ND3300 fluorospectrometer (Thermo Scientific, Canada). The optimal excitation and emission wavelengths of curcumin were set to 420 ± 10 nm and 525 ± 10 nm, respectively. All experiments were performed in duplicate unless otherwise mentioned. The reported relative fluorescence (RF) data were calculated using the standard mathematical equation (RF = F_0_ – F_t_/F_0_), where F_0_ and F_t_ are the original and new fluorescent intensities at 525 nm respectively. Binding affinities of curcumin under the various experimental conditions were calculated by the titration of a varied aptamer concentration with a constant curcumin concentration (0.6 μM) and obtaining saturation binding isotherms. The *K_D_* was determined by applying the Langmuir model [43] through nonlinear regression analysis using the following equation:

(1) $$Y=B_{max}\;\frac{X}{X+\;K_{D}},$$

where *X* is the concentration of aptamer, *Y* is the fluorescent signals at 525 nm, and *Bmax* is the maximum fluorescent signal at 525 nm.

Targets detection: VTD3, BPA, and interfering agents were prepared in pure ethanol (at a concentration of 0.01 M) and further dilutions (to reach mM, μM, nM, and pM concentrations) were made in 0.1 mM NaCl salted water containing 5% ethanol (referred to as stock solutions). The content of ethanol was kept 5% through the whole series of dilutions to ensure sufficient target and curcumin solubility. All of these solutions were made freshly on a daily basis. In order to make different target concentrations for sensing, a given volume from the target stock solution was added to the 0.5 mL solution containing a 10 min pre-incubated 100 nM aptamer with 0.6 μM curcumin in NaCl salted water containing 5% ethanol. The mixture was allowed to react for 15 min, then fluorescence measurements were conducted as noted above. For the detection of VTD3 from blood samples, the previously published extraction protocol by our group [44] was flowed as well as all the additional assessment steps of VTD3 concentrations using HPLC. After n-hexane purification, extraction, and drying steps, the samples were re-dissolved in 0.1 mM NaCl salted water containing 5% ethanol and spiked with the desired VTD3 concentration. All the detection steps were followed as described above. Blood samples were collected from a healthy male. In the case of SYBER Green I based sensor, the same experimental procedure was followed except replacing 0.6 μM curcumin with 5 µM SYBER Green I. The optimal excitation and emission wavelengths of SYBER Green I were set to 495 ± 10 nm and 520 ± 10 nm, respectively. Note: SYBER Green I was received as SGI (10,000×). It was diluted to 500× with in 0.1 M NaCl salted water containing 5% ethanol. Then 20 μM was made freshly based on the Lambert-Beer law [34] in 0.1 M NaCl salted water containing 5% ethanol before use.

Circular dichroism (CD) experiments: Jasco J-815 CD Spectropolarimeter (Easton, MD, USA) instrument was used to measure CD spectra over the wavelength range from 200 to 350 nm with a scanning rate of 2 nm min^−1^. A total volume of 200 µL was used to fill an ultra-thin quartz cell with the test solutions. The concentration of aptamer, VTD3, curcumin, and SYBER Green I in the tested solutions are 0.8 µM, 20 µM, 0.6 µM, and 5 µM respectively.

## 3. Results and Discussion

### 3.1. Curcumin and its Interaction with ssDNAs

Curcumin, 1,7-bis(4-hydroxy-3-methoxyphenyl)-1,6-heptadiene-3,5-dione, is a polyphenol bioactive compound derived from the herbal remedy and dietary spice turmeric [45], the molecular structure is shown in Scheme 1. It has been proven that curcumin possesses diverse pharmacological and biological activities such as; anticancer, antibiotic, anti-inflammatory, and others [45]. Since DNA is the predominant target molecule for emerging synthetic and natural drugs, many studies have characterized the binding mechanism of curcumin to DNA (calf thymus-DNA, AT, and GC alternating heteropolymers) [46,47,48]. These studies concluded that: (1) The DNA curcumin binding could be groove, or intercalative; (2) DNA double helix is essential for curcumin interaction, (3) sodium ions are essential for the DNA–curcumin complex formation, and (4) the formation of the DNA–curcumin complex resulted in an enhancement of light absorption and emission of the curcumin molecule [46,47,48].

Furthermore, curcumin possesses several structural features that make it a favorable and versatile binding probe for DNA. These features include that π–π and van der Waals interactions with aromatic and other hydrophobic entities of DNA can be maximized by the flexible conformation of the two hydrophobic phenyl groups connected by a flexible seven-carbon spacer. Additionally, the hydrophilic groups (hydroxyl, methoxy, ketone, and enol groups) present on the middle and ends of the molecule can contribute to the formation of directed hydrogen-bonding. The seven-carbon spacer contains two α, β-unsaturated carbonyl groups that undergoes keto–enol tautomerism, which is the favorable state for binding with macromolecules including DNA [45,49]. All of these properties encouraged the construction of an effective hypothesis that curcumin is a potential aptasensor probe for small molecules.

### 3.2. Curcumin Interaction with VTD3 Aptamer

**Figure 1:**
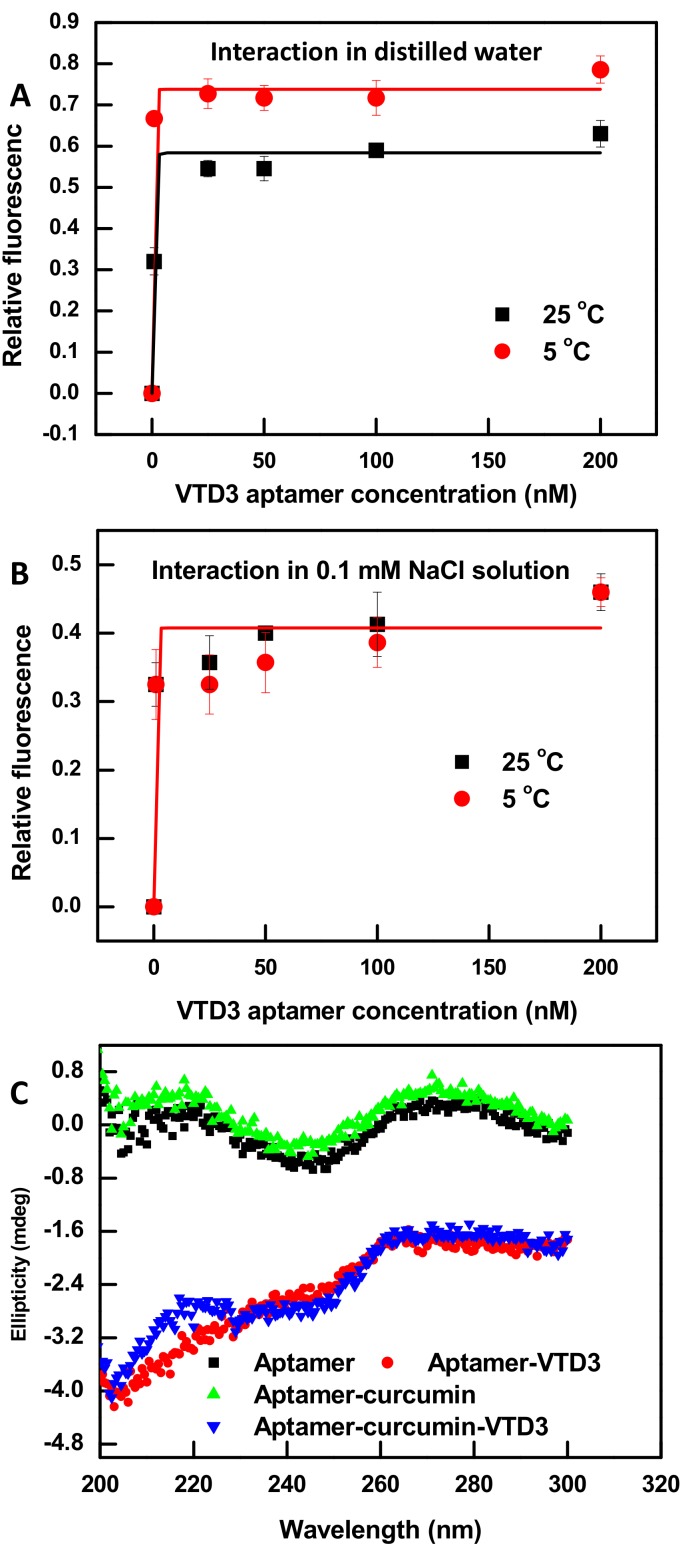
Fluorescence characterization of the interaction of curcumin molecule (0.6 µM) with VTD3 aptamer in (**A**) deionized water and (**B**) 0.1 mM NaCl solution and the effect of temperature on the degree of fluorescent enhancement. Error bars represent standard deviations from two measurements. Lines are non-linear fits of the data for *K_D_* measurements. (**C**) Circular dichroism (CD) measurements of different aptamer samples generated during the construction of the curcumin based sensor and detection of VTD3. The concentration of aptamer, curcumin, and VTD3 are 0.8 µM, 0.6 µM, and 20 µM respectively.

ssDNA aptamers can adopt a variety of secondary and tertiary structures to function their biological target binding role. They constitute of 30–100 nucleotides in length with abundant stem-loop intramolecular base pairing structures [50]. Although curcumin’s binding to synthetic hetero ssDNAs has been reported [46], spectroscopic characterization of its binding to individual aptamers is yet to be demonstrated. Figure 1A,B show fluorescent characterization of the interaction of curcumin molecule (0.6 µM) with increasing concentration of a previously isolated 56-mer VTD3 aptamer [51]. The experiments were conducted in varied sodium ion concentration and temperature conditions. A common feature of the data of these experimental conditions is that the fluorescence of curcumin increased by at least four-folds when it complexes with VTD3 aptamer. As shown in Figure 1A, decreasing the reaction temperature of unsalted deionized water from 25 °C to 5 °C resulted in a higher binding affinity of curcumin to the aptamer, *K_D_* values of 1 nM vs. 0.25 nM respectively. It is well-known that elevated temperatures promote denaturation of the ssDNA secondary structures [52], which are necessary for curcumin binding [46] and abundantly present in the predicted secondary structure of VTD3 aptamer, shown in Figure S1. On the contrary, varying the temperature of 0.1 mM Na ions solutions (Figure 1B) had no effects on the binding affinity of curcumin to the 56-mer aptamer (*K_D_* of 0.7 nM) under different temperatures, due to the stabilized formation of short duplex structures within ssDNA by sodium ions [53]. These observations are consistent with a previous report that sodium ions were essential for binding of curcumin to synthetic hetro ssDNAs [46]. It should be noted that further target sensing experiments should be carried out with the lowest aptamer concentration that gives maximum fluorescent enhancement upon interaction with the curcumin (100 nM) and in salted solutions (0.1 mM sodium ion) to avoid potential errors from temperature variation.

To further explore the binding mode of curcumin and its ability to perturb the 3D structure of the 56-mer aptamer, CD experiments were conducted. CD measurements can be used to study the free conformations of nucleic acid aptamers in-solution, and any subsequent conformational change induced by binding other species [54]. VTD3 aptamer is classified as a random B-form oligonucleotide characterized by a CD spectrum of maximum and minimum ellipticities around 260–280 nm and 240 nm, respectively [54], which is predominantly what is observed in Figure 1C for the free 56-mer VTD3 aptamer, although a second maximum at 210 nm is seen with nearly equal amplitude as the one at 260–280 nm. The positions and amplitudes of these ellipticities are retained unchanged after aptamer-curcumin complex formation, Figure 1C. Thus, curcumin binding does not induce conformational change within the aptamer structure and the interaction can be classified as a groove binding, i.e., insertion of curcumin molecule in the voids within the double helix of the ssDNA.

Additionally, to confirm if curcumin combines with groove structures or intercalates between base pairs, a 10-mer complementary sequence was hybridized to the 56-mer VTD3 aptamer (at the bases 18 to 28) to increase the base pairing in the studied sequence. Independently titrating curcumin with increasing concentrations of ssDNA aptamer and its duplexed structure resulted the same degree of enhancement, shown in Figure S2 in the Supporting Information. The lack of additional enhancement in curcumin fluorescence with the duplexed form of the aptamer rules out the intercalation reaction. It is worth noting that other studies, including [46,48], have reached the same conclusions that curcumin is a groove binding ligand. Additional evidences were obtained by repeating the aptamer duplex structure titration (Figure S2B) and CD experiments (Figure S3C) with the known intercalator dye SYBER Green I. An enhancement in the dye’s fluorescence compared to its fluorescence with the aptamer alone and also a change in the aptamer’s conformation were observed which confirmed the drawn conclusions from this study and others.

### 3.3. Construction of VTD3 Sensor

**Figure 2:**
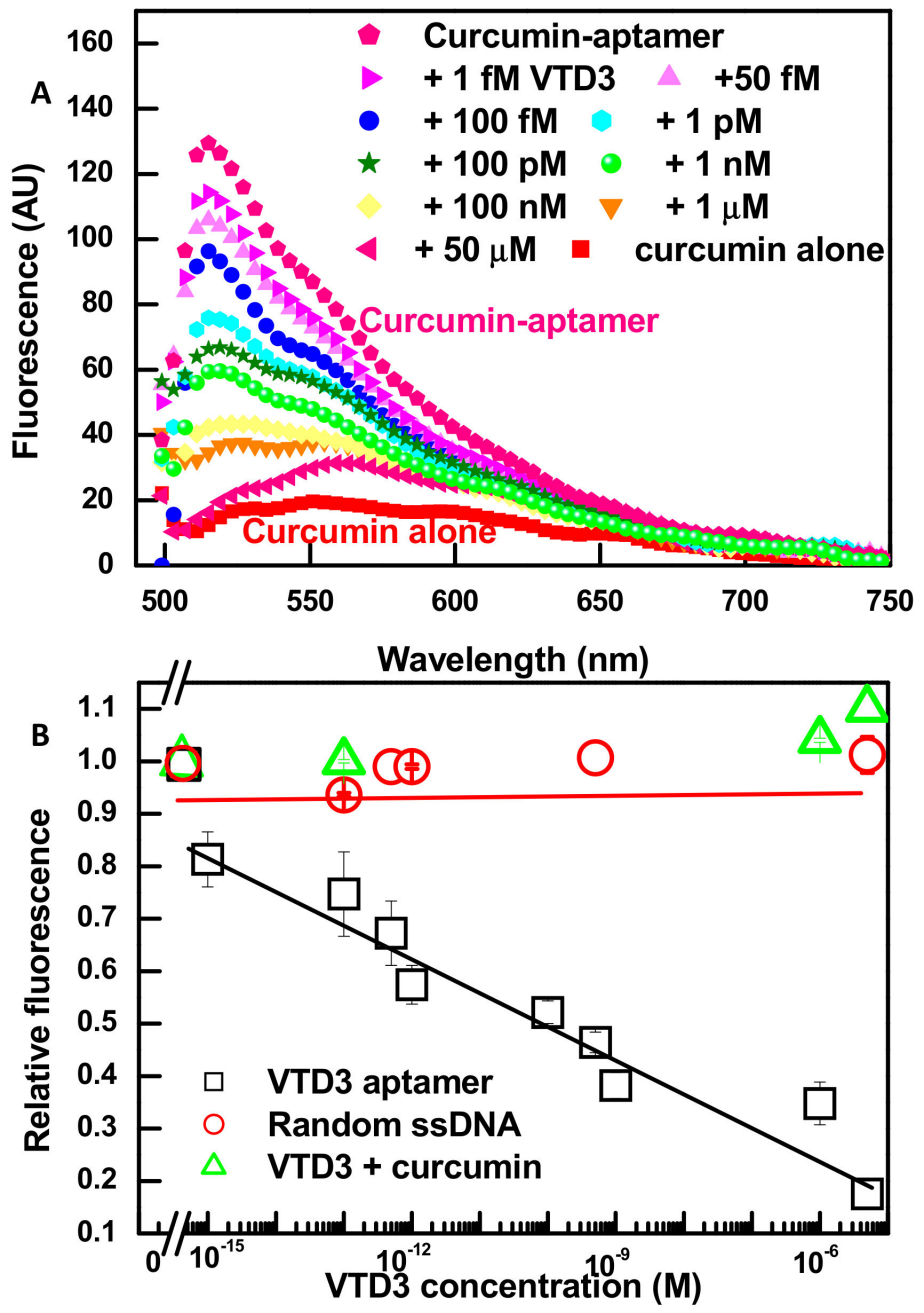
(**A**) Fluorescent spectra showing the fluorescent enhancement upon the aptamer-curcumin complex formation (concentrations of VTD3 aptamer and curcumin are 100 nM and 0.6 µM, respectively) and characterizing the response towards increasing concentrations of VTD3. (**B**) Differential change in fluorescence (relative fluorescence) versus the concentration of VTD3. The response towards VTD3 is also shown for control experiments lacking the VTD3 aptamer (by replacing the aptamer with a random ssDNA or measuring the fluorescence of curcumin exposed to increasing concentration of VTD3). Error bars represent standard deviations from two measurements.

Having established that the association of curcumin molecule into the VTD3 aptamer does not change the aptamer structure, with a binding affinity of 0.7 nM, and is associated with a significant enhancement in its fluorescence, we proceeded to explore the ability of aptamer-curcumin complex to recognize the presence of the target molecule. Figure 2A shows fluorescent spectra of various samples generated throughout the construction of the sensor and the detection of the target. Firstly, an enhancement of nearly five-folds in curcumin’s emission signals is resolved when it is incorporated into the aptamer structure. Secondly, upon incubation of the aptamer-reporter complex with increasing concentrations of VTD3 (1 fM up to 1 µM), the fluorescence decreases sequentially approaching the signal of curcumin alone. Figure 2B shows a well-correlated fluorescence response (reported as relative change) with increasing VTD3 concentrations. The lowest detected concentration of VTD3 is 1 fM and the sensor operates over four orders of magnitude range of target concentration. The sensor is highly robust where the error bars in Figure 2B represent the standard deviation of two experiments. It was confirmed that the VTD3 detection presented in Figure 2 resulted from the specific conformational change of the target binding by conducting two control experiments: (A) Repeating the sensing experiments with a random ssDNA and (B) exposing the curcumin directly to increasing concentrations of VTD3. As shown in Figure 2B, the observed VTD3 sensitivity was not retained when the aptamer was replaced with a randomized sequence that lacks the VTD3 binding domain and when curcumin was mixed with VTD3, which verify our observations.

In this study, it has been established that aptamer probe can form groove structures capable of binding curcumin, which leads to enhanced off green fluorescence. While in the presence of the target, the aptamer probe binds to its target preferentially, leading to significant conformational change within the aptamer and disruption of the curcumin’s binding sites. This promotes the dissociation of curcumin molecule and results in the disappearance of the enhanced fluorescence. To demonstrate the feasibility of this hypothesis, CD experiments were conducted. Figure 1C shows that VTD3 recognition resulted in a CD spectrum with bands entirely different in shape and amplitudes than those of free aptamer. Similar CD spectrum was recorded when curcumin was additionally present in the detection mixture, which confirms the above mentioned assumption that target recognition induces displacement of curcumin. Furthermore, the CD results are consistent with our fluorescent experiments presented in Figure 2, where target recognition reverses the fluorescent enhancement that accompanies curcumin binding.

To the best of our knowledge, the 1 fM detection limit has not been reported for the labeled or label-free fluorescent aptasensor for small molecules [40]. The remarkable sensitivity of the reported detection design could be attributed to two factors: (A) High affinity of the aptamer (*K_D_* of 11 nM reported by [51]) accompanied with substantial target-induced conformational change, and B) groove based binding (*K_D_* of 0.7 µM) of the curcumin molecule to ssDNA aptamer. With these characteristics, perceptible signal transduction is surprisingly achieved at orders of magnitude lower target concentration than the *K_D_* value of aptamer.

Repeating the sensing steps with the intercalator dye SYBER Green I achieved only a 1 nM detection limit and a limited dynamic range (Figure S3A), which demonstrates the favorable comparison for the developed curcumin sensor. Additionally, due to the different interaction mode between aptamer and SYBER Green I, target detection was found to produce increased fluorescent signal. It could be explained as the following: In the absence of the target, the aptamer probe can partially form a double strand stem, which binds to SYBER Green I. However, in the presence of the target, the aptamer forms more double strand structures and results in a further enhancement in the fluorescence of the intercalator dye. These speculations were confirmed by the CD experiments presented in Figure S3C.

### 3.4. Specificity of VTD3 Sensor

**Figure 3:**
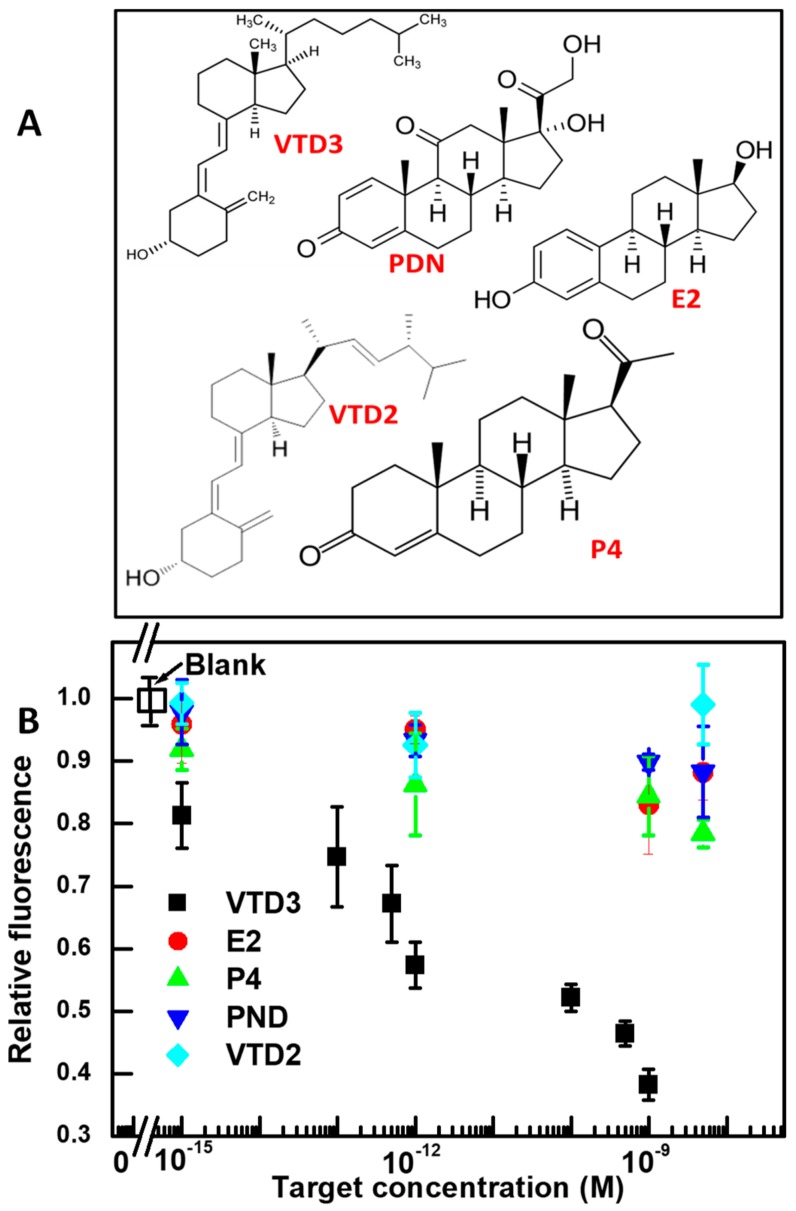
(**A**) Molecular structures of VTD3 and the interfering targets used to evaluate the specificity of the aptasensors developed in the study. (**B**) Differential change in fluorescence (relative fluorescence) vs. concentration of VTD3 and interfering molecules. The concentrations of VTD3 aptamer and curcumin are 100 nM and 0.6 µM, respectively. Error bars represent standard deviations from two measurements.

Using the 56-mer VTD3 binding aptamer sequence, we repeated the sensing steps with structurally similar molecules that either co-exist with VTD3 in biological samples (VTD2, P4, and E2), or could trigger a similar aptamer conformational switch when encountered (PDN). The molecular structures of these agents is shown in Figure 3A. As shown in Figure 3B, the aptamer is remarkably specific, producing only a baseline response to the potential interfering molecules examined, even at concentrations as high as 1 µM. These findings show that the fluorescent signal results specifically from the aptamer sequence binding with its target VTD3. The observed specificity is achieved as a result of the non-disrupted 3D structure of aptamer upon binding with curcumin established above, as well as the intrinsic specificity of this aptamer sequence. The specificity results reported here are in agreement with those reported previously, when the same aptamer sequence and interfering molecules were examined using a colorimetric gold aggregation sensor [44]. Additionally, the excellent specificity found by this study is broadly consistent with the study of Lee et al. when other interfering molecules were examined [51].

### 3.5. Detection of VTD3 in Blood Samples

**Figure 4:**
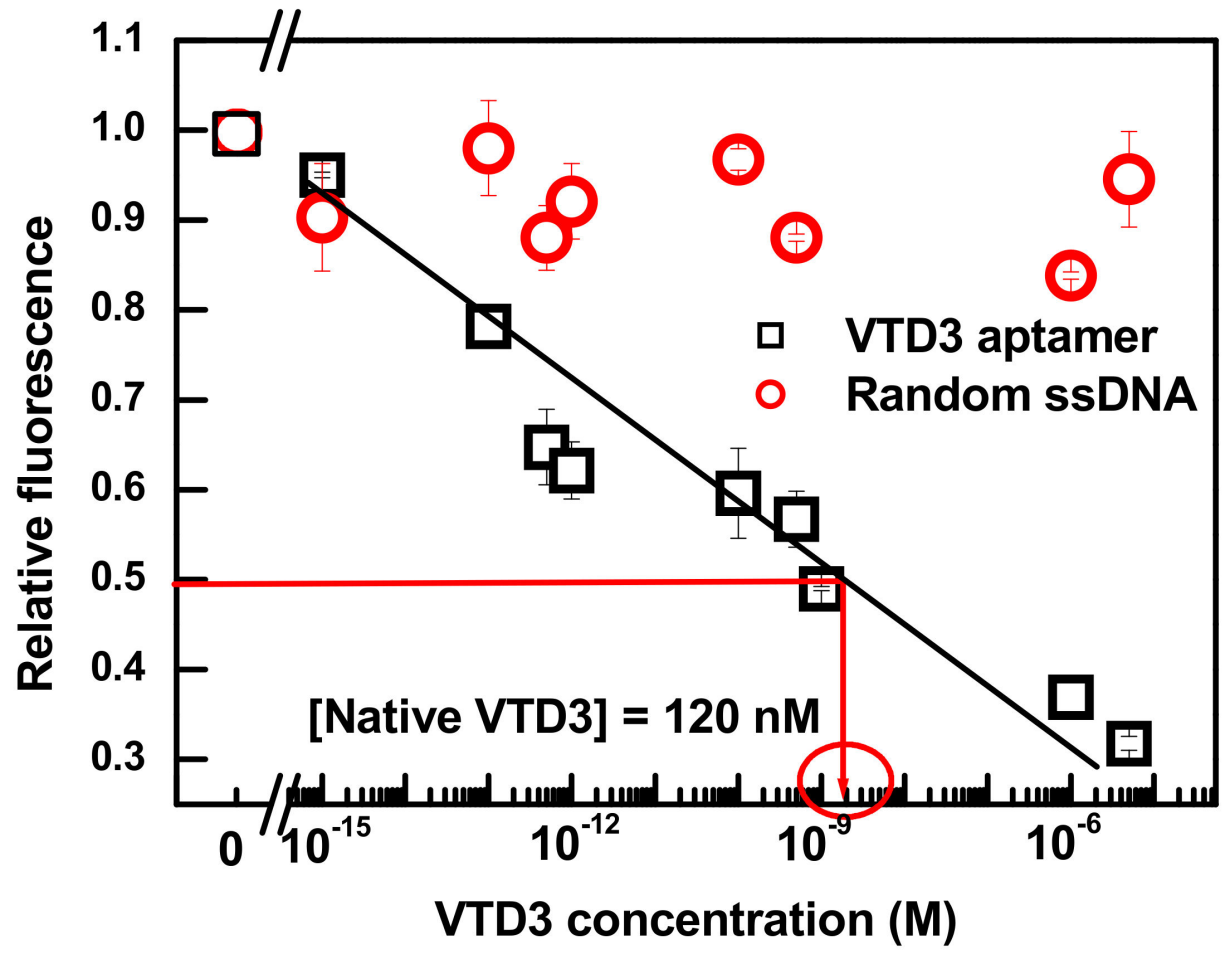
Differential change in fluorescence (relative fluorescence) vs. concentration of VTD3 extracted from whole blood sample. The response towards VTD3 is also shown for a control experiment by replacing VTD3 aptamer with a random 70-mer aptamer. The concentrations of VTD3 aptamer and curcumin are 100 nM and 0.6 µM, respectively. Error bars represent standard deviations from two measurements.

Having demonstrated a label-free sensing platform with 1 fM detection level and excellent specificity, we proceeded to examine its application in a more challenging physiological fluid of blood. The curcumin fluorescent sensor is based on optical detection, and thus is susceptible to interference from colored or turbid samples like blood. Such a limitation can be overcome by a simple n-hexane extraction that allows the isolation of the desired VTD3 molecule, along with other hydrophobic small molecules, from the rest of the blood matrix in a short time, less than 10 min [44]. As shown in Figure 4, the curcumin based sensor delivers fluorescent signals for spiked blood samples similar to those observed when detecting VTD3 in buffer (Figure 2) and with a detection limit as low as 200 fM, defined as S/N = 3. The sensor lacked this specific response when the VTD3 aptamer was replaced by a random ssDNA sequence, as shown in Figure 4, refer to fluorescent spectra in Figure S4

Although the detection limit of the sensor is diminished compared to its performance in buffer, the 200 fM level of detection is approximately five orders of magnitude lower than the recorded concentration of VTD3 in human blood, ranges between 50 nM to 150 nM [55]. The calibration curve of the sensor response to the spiked blood samples was used to directly determine VTD3 from blood samples. The resolved VTD3 concentration, indicated by the red lines in Figure 4, was 120 nM, which falls within the normal VTD3 level of a healthy individual [55]. The determination of VTD3 from blood samples by curcumin based sensor was consistent with what was reported by our group using high performance liquid chromatography and gold based colorimetric sensor, 120 nM and 115 respectively [44].

Figure 4 shows that a simple and reliable label-free aptasensor can be constructed by simply binding the natural dye curcumin into the desired aptamer and monitoring the fluorescent signals upon exposure to the test sample. The level of detection is over five orders of magnitude lower than our previous colorimetric VTD3 sensor based on a dispersion of AuNPs coated with exactly the same aptamer sequence [44]. Indeed, the reported level of detection is at least six orders of magnitude lower than the routinely used liquid chromatography coupled with mass spectrometry (LOD = 10 nM) [56], antibody-based-ELISA assay (LOD = 5 nM), and antibody-based-chemiluminescence assay (LOD = 18 nM) [57]. Additionally, our aptasensor platform provides orders of magnitude lower detection limit than other label-free fluorescent aptasensors including the detections of ochratoxin A (LOD = 9 nM) [36], ATP (LOD = 1.45 µM) [37], L-argininamide (LOD = 2.5 µM) [38], and tetracycline (LOD = ~65 nM) [39]. Additionally, the four orders of magnitude dynamic range reported in this study was significantly wider than all reported label-free fluorescent aptasensors, with most only covering a narrow range of target concentration.

### 3.6. Generality of the Sensor

**Figure 5:**
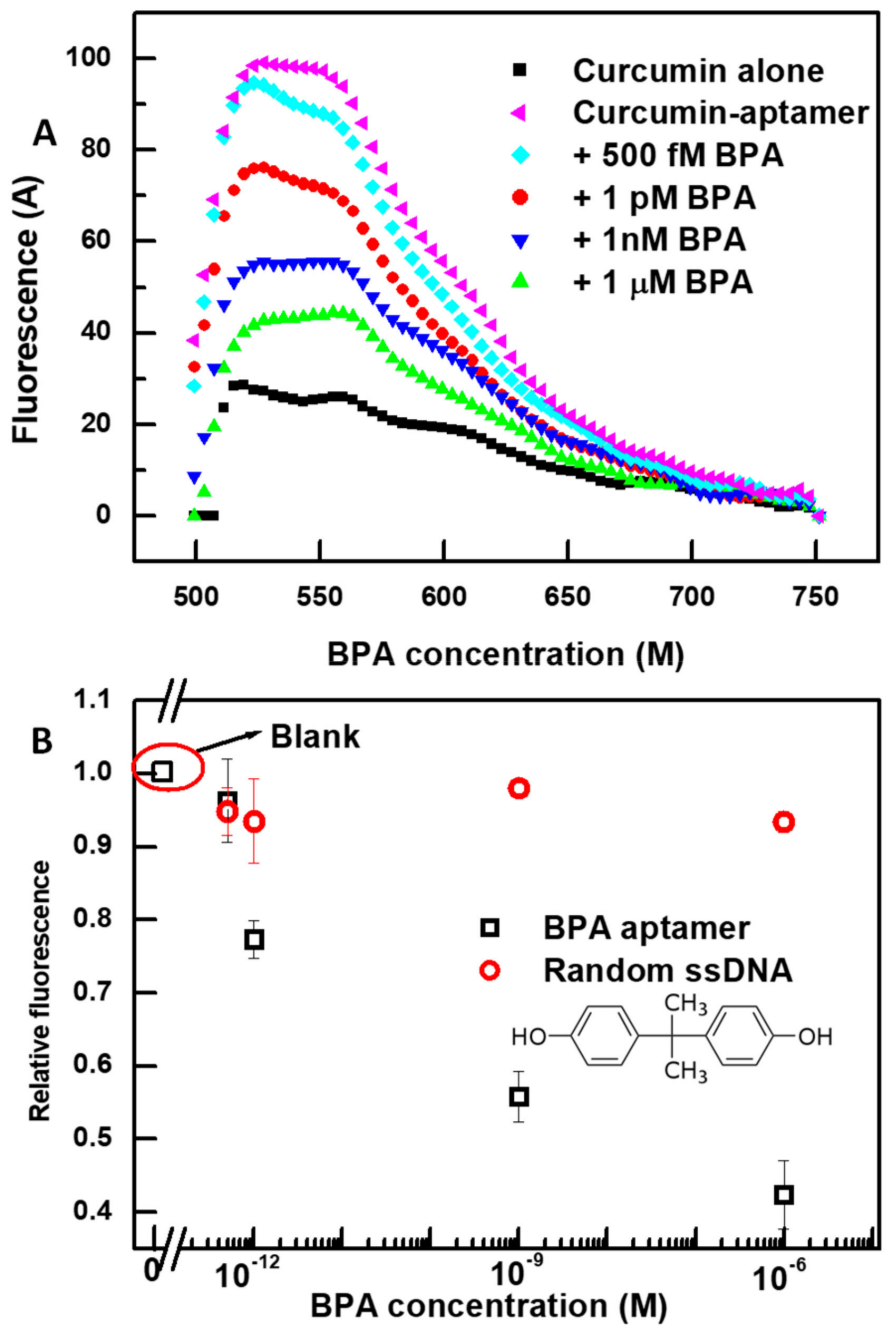
(**A**) Fluorescent spectra of the detection of BPA at increasing concentrations using the specific 75-mer BPA aptamer and the developed curcumin-based signal-off fluorescent sensor. The same conditions of VTD3 sensors were used in this case: 0.6 µM of curcumin, 100 nM of BPA aptamer, and detection in 0.1 mM NaCl solution. (**B**) Differential change in fluorescence (relative fluorescence) versus the concentration of BPA. The response towards BPA is also shown for a control experiment with a random 70-mer ssDNA. Error bars represent standard deviations from two measurements. The molecular structure of BPA is shown in the figure.

We confirmed that the proposed scheme could be applied to detect other low molecular weight targets, as long as the desired aptamer is available. We used a recently published aptamer sequence for the target BPA [58]. Firstly, we made sure that curcumin interaction with the 75-mer BPA aptamer resulted in a fluorescent enhancement as shown in Figure 5A. Similarly to VTD3 aptamer, the secondary structure of BPA aptamer (presented in Figure S1) shows that the sequence contained multiple stem-loop structures, which could act as potential curcumin binding centers. By following the same protocol used for the VTD3 detection and upon exposure to BPA, the sensor produced fluorescent signals similar to those observed when detecting VTD3. Figure 5B shows the lowest detected concentration of BPA was 500 fM and the sensor operated over a wide dynamic range. The sensor lacked this specific response when the BPA aptamer was replaced by a random 70-mer sequence. It should be noted that the sensor delivered more than seven orders of magnitude better sensitivity than when the same aptamer was applied in a lateral flow aptasensor [58]. Additionally, the presently proposed sensing platform for BPA either provided better or the same order of magnitude detection limit compared to other sensing aptasensors for BPA when a different BPA aptamer was implemented [59]. These sensors include electrochemical (LOD = 20 pM) [60], labeled fluorescent (LOD = ~0.744 pM), and colorimetric sensor based on gold aggregation (LOD = ~0.4 nM) [61].

## 4. Conclusions

We demonstrated that the binding of the natural dye curcumin to ssDNA aptamers was groove based binding with binding constant in the nM concentration level. The coupling of the dye to nucleic acids resulted in a substantial enhancement in the off-green emission spectra. Such a non-covalent binding mode of the dye was found to be reversible by the conformational change induced by the small target recognition. Perceptible and well-correlated fluorescent signals were recorded upon target binding. The phenomenon was exploited in the demonstration of a new sensor platform for small molecules using only the unmodified aptamer sequences and curcumin molecule. Unlike previous label-free aptasensors that probe conformational changes with the strongly bound intercalators, the present sensor resolved pronounced changes in fluorescence with a femtomolar level VTD3 concentration, operated over a wide dynamic range, and showed excellent discrimination against potential interfering agents, and robust operation in extracted samples from the complex physiological fluid of human blood. The combination of these remarkable sensing properties were proven to be general and were demonstrated with the BPA aptamer. The reported curcumin based label-free strategy holds promising potential for the development sensors targeting large targets, routine characterizations needed post to SELEX selection rounds, and provides a novel fluorophores for multiplex targets detection when used with other dyes.

## Supplementary Materials

The following are available online at https://www.mdpi.com/1424-8220/19/19/4181/s1, Figure S1: secondary structures of VTD3 and BPA aptamers, Table S1: sequences used in this study, Figure S2: interaction of SYBER Green I and curcumin molecules with duplexed strand of the VTD3 aptamer, Figure S3: fluorescent signals and CD measurements of VTD3 sensor based on SYBER Green I, and Figure S4: fluorescent spectra of VTD3 detection in extracted blood samples.
