# Supplementary material for: Label-Free Fluorescent Aptasensor for Small Targets via Displacement of Groove Bound Curcumin Molecules
Source: Sensors (Basel). 2019 Sep 26;19(19):4181. doi: 10.3390/s19194181 (PMC6806071; doi:10.3390/s19194181)
Supplement: Supplementary file 1 [file sensors-19-04181-s001.pdf]

Supplementary Materials:

Label-Free Fluorescent Aptasensor for Small Targets via Displacement of Groove Bound Curcumin Molecules

Table S1. Sequences used in this study.

Name	Sequence
**56-mer VTD3 aptamer** [1]	5′AGCAGCACAGAGGTCATGGGGGGTGTGACTTTGGTGT GCCTATGCGTGCTACGGAA-3′
**10-mer VTD3 aptamer complementary sequence**	CCCCCACACTGAAACCACAC
**75-mer BPA aptamer** [2]	ATACGAGCTTGTTCAATAGGAAATCACGATTAGGTCCT CCGTCTGTGTGCGGTTGTGGTGATAGTAAGAGCAATC
**Random ssDNA (70-mer)**	5′AGGCCTAAGGGCATAATTAGCTCGAGCTCGAAAGGG GTTATATGATGATTTGAATTCATGGGGCCCGACT-3′

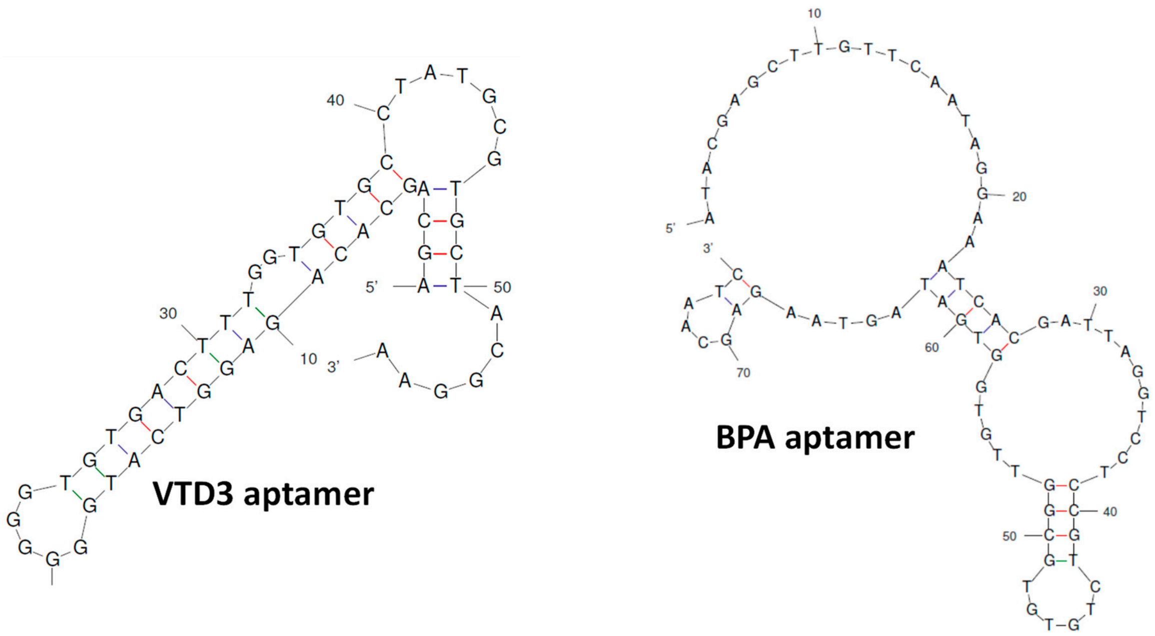

Figure S1. Shows the secondary structures of the 56-mer VTD3 aptamer [1] and 75-mer BPA aptamer [2]. Secondary structures were determined using the web-based tool m-fold with the free-energy minimization algorithm.

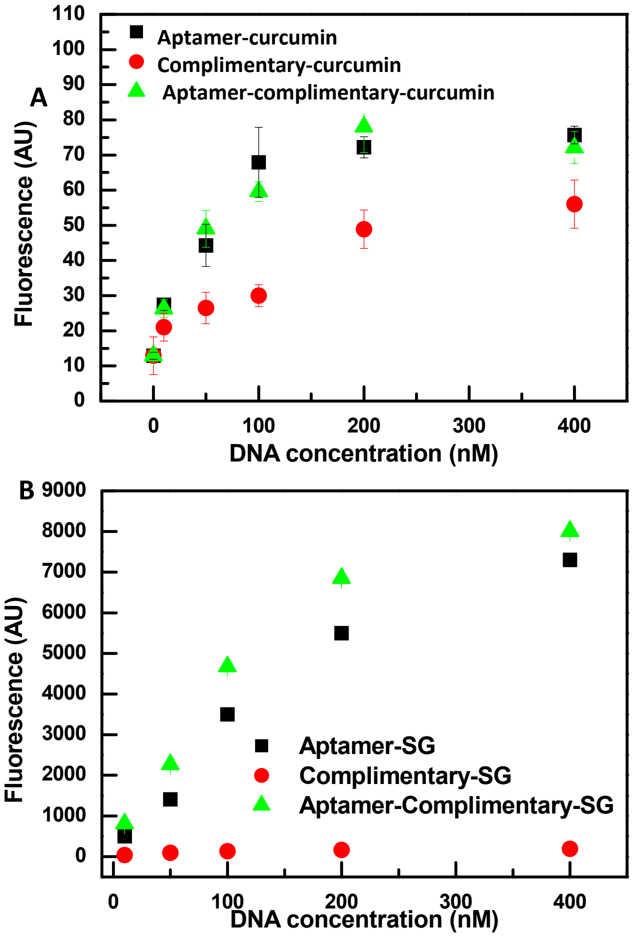

Figure S2. interaction of curcumin **(A)** and SYBER Green I **(B)** with VTD3 aptamer, 10-mer complementary sequence, and VTD3 aptamer-complementary sequence duplex structure for determining of the binding mechanism between curcumin and the aptamer.

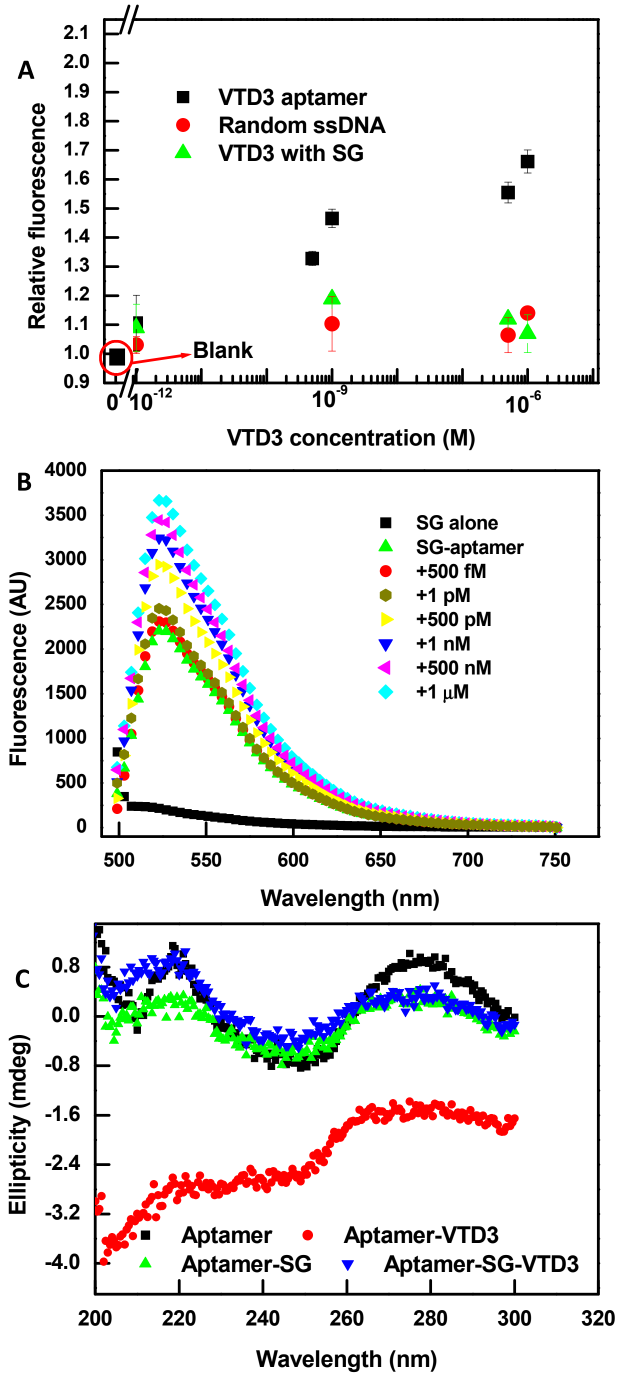

Figure S3. (A) differential change in SYBER Green I fluorescence (relative fluorescence) vs. increasing concentrations of VTD3. The response towards VTD3 is also shown for two control experiments: 1) replacing VTD3 aptamer with a random 70-mer aptamer and 2) exposing increasing concentrations of VTD3 to SYBER Green I. The concentrations of VTD3 aptamer and SYBER Green I are 100 nM and 5 μM, respectively. Error bars represent standard deviations from two measurements. **(B)** shows fluorescence spectra of the detection of VTD3 at increasing concentrations using the specific 56-mer VTD3 aptamer and the SYBR Green I based fluorescence sensor. **(C)** CD measurements of different aptamer samples generated during the construction of the SYBER Green I based sensor and the detection of VTD3. The concentration of aptamer, SYBER Green I, and VTD3 are 5 μM, 1 μM, and 20 μM respectively.

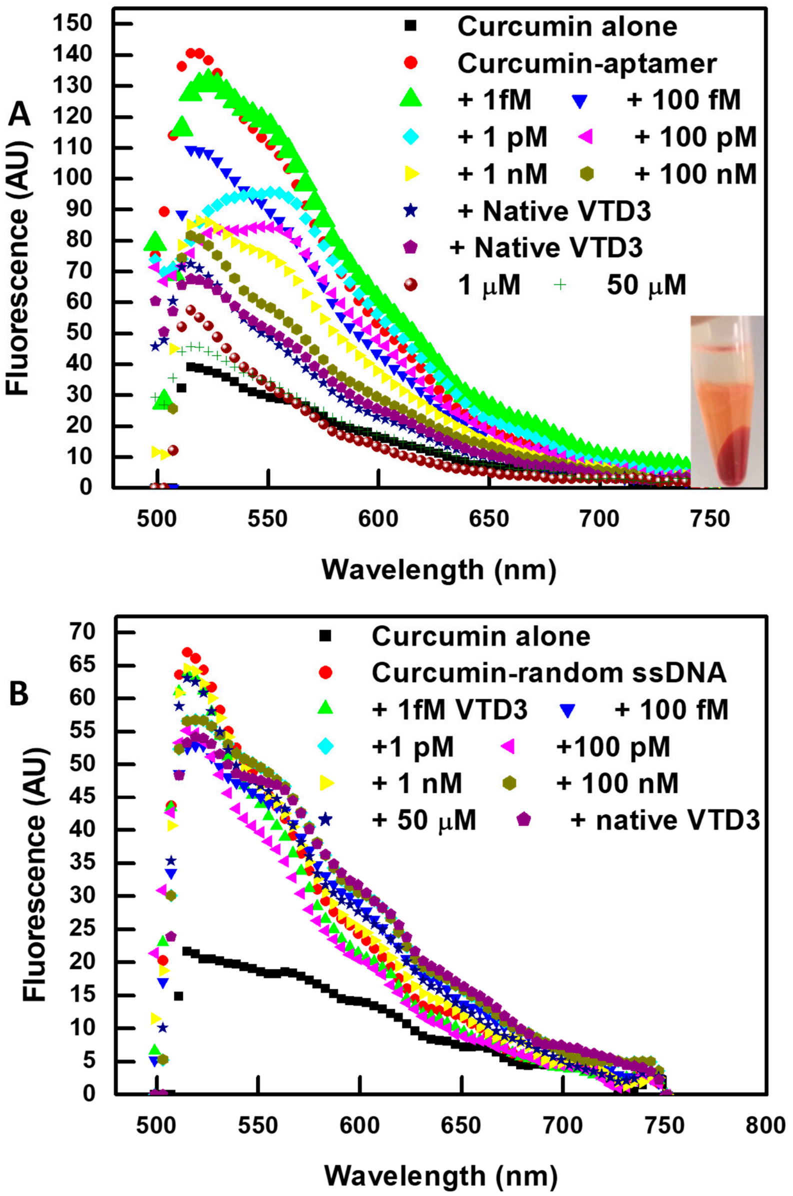

Figure S4. **(A)** fluorescent spectra of VTD3 the detection of VTD3 in extracted blood samples using VTD3 binding aptamer. (**B)** fluorescent spectra of the detection of VTD3 in extracted blood samples using random ssDNA. The same conditions of VTD3 sensor in buffer were used in this case: 0.6 μM curcumin concentration, 100 nM concentration of VT3 aptamer, and detection was conducted in 0.1 mM NaCl solution. A photo of blood sample after incubation with n-hexan and centrifugation is presented in the figure.

References

1. Lee, B.H.; Nguyen, V.T.; Gu, M.B. Highly sensitive detection of 25-HydroxyvitaminD3 by using a target-induced displacement of aptamer. *Biosens. Bioelectron.* **2017**, *88*, 174–180.

2. Alsager, O.A.; Kumar, S.; Hodgkiss, J.M. Lateral Flow Aptasensor for Small Molecule Targets Exploiting Adsorption and Desorption Interactions on Gold Nanoparticles. *Anal. Chem.* **2017**, *89*, 7416–7424.
